# Targeting Cathepsin L in Cancer Management: Leveraging Machine Learning, Structure-Based Virtual Screening, and Molecular Dynamics Studies

**DOI:** 10.3390/ijms242417208

**Published:** 2023-12-07

**Authors:** Abdulraheem Ali Almalki, Alaa Shafie, Ali Hazazi, Hamsa Jameel Banjer, Maha M. Bakhuraysah, Sarah Abdullah Almaghrabi, Ahad Amer Alsaiari, Fouzeyyah Ali Alsaeedi, Amal Adnan Ashour, Afaf Alharthi, Nahed S. Alharthi, Farah Anjum

**Affiliations:** 1 Department of Clinical Laboratory Sciences, College of Applied Medical Sciences, Taif University, Taif 21944, Saudi Arabia; almalki@tu.edu.sa (A.A.A.); dr.alaa.shafie.tu@gmail.com (A.S.); h.banjer@tu.edu.sa (H.J.B.); mbakhuraysah@gmail.com (M.M.B.); ahadamer@tu.edu.sa (A.A.A.); fouzeyyah@tu.edu.sa (F.A.A.); a.awwadh@tu.edu.sa (A.A.); 2 Department of Pathology and Laboratory Medicine, Security Forces Hospital Program, Riyadh 11481, Saudi Arabia; ahazazi@sfh.med.sa; 3 Department of Medical Laboratory Sciences, Faculty of Applied Medical Sciences, King Abdulaziz University, Jeddah 21589, Saudi Arabia; saalmaghrabi@kau.edu.sa; 4 Center of Innovations in Personalized Medicine (CIPM), King Abdulaziz University, Jeddah 21589, Saudi Arabia; 5 Department of Oral and Maxillofacial Surgery and Diagnostic Sciences, Faculty of Dentistry, Taif University, Taif 21944, Saudi Arabia; a.a.ashour@tu.edu.sa; 6 Department of Medical Laboratory Sciences, College of Applied Medical Sciences in Al-Kharj, Prince Sattam Bin Abdulaziz University, Al-Kharj 11942, Saudi Arabia; n.alharthi@psau.edu.sa

**Keywords:** cathepsin L, cancer, natural compounds, machine learning, drug-likeness

## Abstract

Cathepsin L (CTSL) expression is dysregulated in a variety of cancers. Extensive empirical evidence indicates their direct participation in cancer growth, angiogenic processes, metastatic dissemination, and the development of treatment resistance. Currently, no natural CTSL inhibitors are approved for clinical use. Consequently, the development of novel CTSL inhibition strategies is an urgent necessity. In this study, a combined machine learning (ML) and structure-based virtual screening strategy was employed to identify potential natural CTSL inhibitors. The random forest ML model was trained on IC_50_ values. The accuracy of the trained model was over 90%. Furthermore, we used this ML model to screen the Biopurify and Targetmol natural compound libraries, yielding 149 hits with prediction scores >0.6. These hits were subsequently selected for virtual screening using a structure-based approach, yielding 13 hits with higher binding affinity compared to the positive control (AZ12878478). Two of these hits, ZINC4097985 and ZINC4098355, have been shown to strongly bind CTSL proteins. In addition to drug-like properties, both compounds demonstrated high affinity, ligand efficiency, and specificity for the CTSL binding pocket. Furthermore, in molecular dynamics simulations spanning 200 ns, these compounds formed stable protein-ligand complexes. ZINC4097985 and ZINC4098355 can be considered promising candidates for CTSL inhibition after experimental validation, with the potential to provide therapeutic benefits in cancer management.

## 1. Introduction

Cathepsins are proteases found in lysosomes classified into cysteine, aspartate, and serine cathepsins, depending on their active site [1]. Cysteine cathepsins (CTS) are found in a wide range of organisms, spanning from prokaryotes to mammals, and exhibit a conserved cysteine residue within their active site. These enzymes are essential for degrading proteins internalized by lysosomes and are active at pH levels ranging from neutral to acidic [2]. They also aid in the breakdown of intracellular proteins, the activation of inactive enzyme precursors, and the remodeling of the extracellular matrix [3,4]. CTS serve an important role in tissue homeostasis and contribute to processes such as immune response, apoptosis, and development under normal physiological settings [5]. However, their expression, location, and activity changes have been linked to various clinical diseases, including cancer progression [6,7]. Ectopic cysteine cathepsin expression is frequently associated with a poor prognosis [8]. The number of cathepsin classes in humans has increased from the previously recognized 11 to 15. These include the classes A, B, C, D, E, F, G, H, L, K, O, S, V, X, W, and Z [9].

Cathepsin L (CTSL) functions both intracellularly and extracellularly. The acidic environment created in cancer cells by anaerobic glycolysis promotes the activity of CTSL [10,11], which actively degrades collagen, fibronectin, and laminin [12]. CTSL is normally confined within lysosomes in healthy physiological states. However, in the context of tumors, changes in expression levels and translocation pathways can lead to CTSL secretion. Studies on animal models of pancreatic cancer have demonstrated that CTSL and cathepsin S enzymes are released from cancer cells and act on the extracellular domain of cell adhesion molecules present on the surface of cancer cells [13]. CTSL’s ability to degrade E-cadherin (a cell–cell adhesion molecule) contributes to cancer cells’ invasive function and metastatic capacity [14]. CTSL expression increases in various cancers, including glioma, melanoma, pancreatic, breast, and prostate carcinoma [15]. Given the importance of CTSL in tumor cell dissemination, there is a pressing demand to develop novel CTSL inhibition strategies. Several CTSL inhibitors have been identified, including SID 26681509, quarterhydrate, and Z-FY-CHO [16]. While these inhibitors show CTSL specificity, it is important to note that they are synthetic and not derived from natural sources. Because of their synthetic origin, they may have potential side effects when used. This highlights the importance of investigating natural compounds as potential CTSL inhibitors in order to alleviate concerns about side effects and improve the translational potential of CTS-targeted therapeutic interventions.

The drug development process is characterized by its interdisciplinary nature, significant costs, and time requirements. Over the last two decades, scientific progress has transformed the approach to pharmaceutical research in generating new bioactive molecules [17]. Notably, advances in computational techniques and parallel hardware support have made it easier to use in silico methods, particularly the structure-based drug design approach. These methods have accelerated various stages of the drug development process, including target identification via identifying initial active compounds and subsequent optimization of lead compounds [18]. This study employed in silico strategies, including a combination of machine learning (ML) and structure-based screening of natural compounds, to find natural CTSL inhibitors for combating cancer.

## 2. Results and Discussion

**Figure 1:**
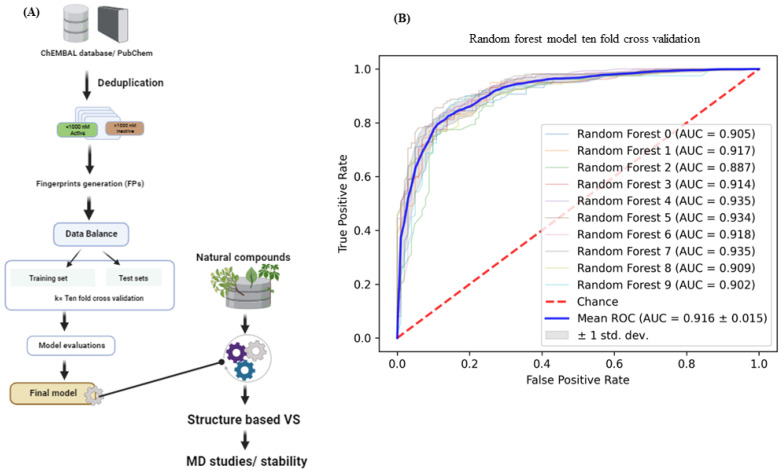
Workflow of study (**A**), and evaluation of the train RF machine learning model for CTSL protein (**B**).

The CHEMBL database was used to obtain compound activity data against the CTSL enzyme. The dataset’s redundancy was examined, and all duplicate compounds were removed. Compounds with IC_50_ values less than 1000 nM were considered active, while those with IC_50_ values greater than 1000 nM were considered inactive. We confirmed 2000 active molecules and 1278 inactive molecules using these criteria. The Morgan fingerprints (1024) have been calculated for the active/inactive molecules. Following vectorization, the random forest (RF) classification model was trained to differentiate between active and inactive molecules. ROC curves from 10-fold cross-validation were plotted. Figure 1A depicts the study’s workflow. The AUC mean values of the RF model were estimated at 0.91 (Figure 1B).

Following that, the RF train model was used to screen the Biopurify and Targetmol natural compound libraries, with the top hits selecting them for further SBVS. A total of 149 compounds with prediction scores >0.6 were identified. These compounds were then used in structure-based virtual screening (SBVS) to selectively interact with the CTSL protein’s active site. In this study, the positive control was AZ12878478. The binding energies that exceeded those of the control compound were chosen for further interaction analysis, which included both two-dimensional (2D) and three-dimensional (3D) interactions. SBVS yielded thirteen hits with higher affinity, as determined by binding energy, in comparison to the positive control (Table 1). Table 1 presents the RF prediction scores and binding affinities of the 13 compounds. Further physicochemical and molecular properties encompass the molecular weight, the number of rotatable bonds, as well as the number of hydrogen bond donors and acceptors.

**Figure 2:**
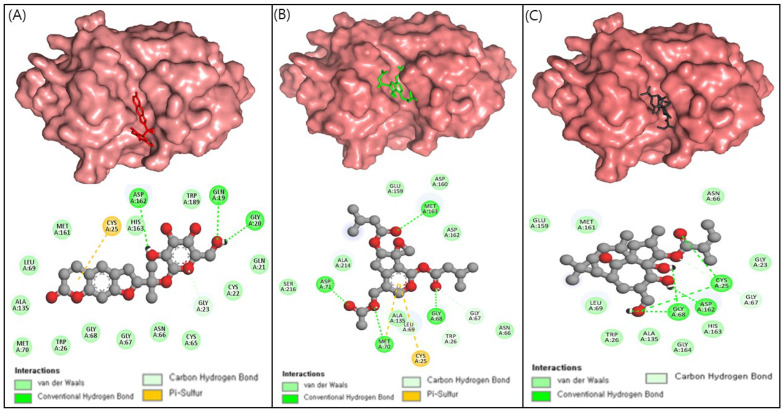
3D and 2D interaction of ZINC4097985 (red) (**A**), ZINC4098355 (green) (**B**), and control compound (black) (**C**) in the CTSL protein binding pocket.

The top two compounds, ZINC4097985, and ZINC4098355, as well as the positive control, were subjected to detailed interaction analysis with the CTSL active site residues. ZINC4097985 was found to interact with Gln19, Gly20, Gln21, Cys22, Gly23, Cys25, Trp26, Cys65, Asn66, Gly67, Gly68, Leu69, Met70, Ala135, Met161, Asp162, His163, and Trp189 residues of CTSL protein. The Gln19, Gly20, and Asp162 residues were H-bonded with ZINC4097985 (Figure 2A). Furthermore, ZINC4098355 interacted with Cys25, Trp26, Asn66, Gly67, Gly68, Leu69, Met70, Asp71, Ala135, Glu159, Asp160, Met161, Asp162, Ala214, and Ser216 residues of CTSL protein. The Gly68, Met70, and Met161 residues were H-bonded with ZINC4098355 (Figure 2B).

The co-crystal inhibitor was found to interact with Gly23, Cys25, Trp26, Asn66, Gly67, Gly68, Leu69, Ala135, Glu159, Met161, Asp162, His163, and Gly164 residues of CTSL protein (Figure 2C). Notably, among these residues, Cys25, Trp26, Asn66, Gly67, Gly68, Leu69, Ala135, Met161, and Asp162 were found to be common interaction residues for the co-crystal inhibitor and the hit compounds (ZINC4097985 and ZINC4098355) (Figure 2A–C). This observation suggests that the hit compounds ZINC4097985 and ZINC4098355 bind to the same binding pocket on the CTSL protein as the co-crystal inhibitor.

CTSL has an electrophilic center that can act as a thiol trap for the active-site cysteine (Cys-25), making it an ideal target for covalent inhibitors. CTSL inhibitors currently use electrophilic moieties such as epoxides, vinyl sulfones, fluoromethyl ketones, and diazomethyl ketones to form an irreversible covalent linkage with CTSL [19,20,21]. Nonetheless, the use of irreversible inhibitors in drug candidate development raises significant safety concerns due to the potential difficulties associated with dissociation. Nitrile-functionalized inhibitors have been classified as covalent and reversible inhibitors due to the capacity of nitriles to selectively bind sulfur atoms, leading to the formation of thioimidazole bonds that exhibit hydrolytic properties over a period of time [22,23]. Importantly, the identified natural compounds in this study (ZINC4097985 and ZINC4098355) have demonstrated interactions with CTSL’s Cys-25 residue, implying that these compounds could be potent CTSL inhibitors.

The evaluation of adsorption, distribution, metabolism, excretion, and toxicity (ADMET) properties holds significant importance in the realm of small-molecule drug discovery and therapeutics. It is important to acknowledge that a multitude of clinical trials have encountered obstacles due to inadequacies in these characteristics. Although early-stage ADMET profiling is considered optimal, the execution of experimental evaluations is often hindered by the high costs involved and the limited availability of data. The ADMET properties of the top two compounds were predicted using online tools (https://ai-druglab.smu.edu/admet, accessed on 10 July 2023) (Table 2) [24]. The anticipated values of diverse molecular properties for these two compounds fell within an acceptable range, suggesting their potential as lead molecules.

**Figure 3:**
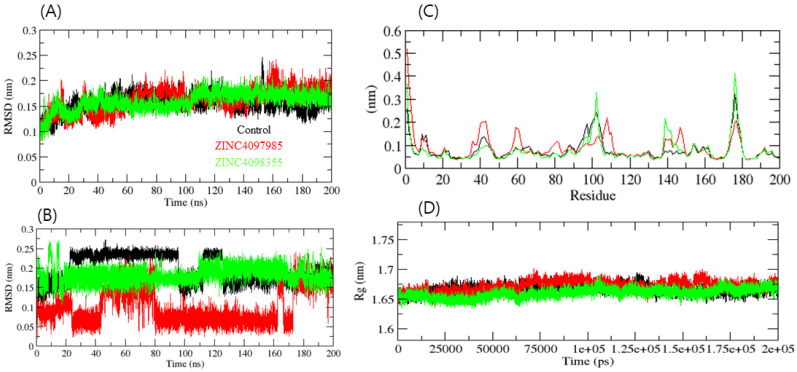
Complex structural stability studies. Protein backbone RMSD (**A**), ligand RMSD plot (**B**), RMSF of protein (**C**), and Rg of the complexes (**D**).

Finally, the docked complexes of these two compounds (ZINC4097985 and ZINC4098355) and the control were subjected to 200 ns of MD simulation. MD simulations of the docked complexes were performed to assess their binding stability. The root mean square deviation (RMSD) serves as a metric for assessing protein stability, with smaller deviations indicating a higher degree of stability in the protein structure. CTSL-control, CTSL-ZINC4097985, and CTSL-ZINC4098355 had average RMSD values of 0.18, 0.21, and 0.15 nm, respectively. The RMSD plot exhibits that CTSL-ZINC4098355 and the CTSL-control complex were more stable than CTSL-ZINC4097985. The bound structure of the CTSL-ZINC4097985 complex exhibited a high deviation from the initial conformation, indicating that CTSL’s catalytic pocket formed a stable interaction with the screened compound. Furthermore, the ligand RMSD exhibits that the control and ZINC4098355 demonstrated high deviation, while the ZINC4097985 complex showed low deviation (Figure 3A,B).

The average fluctuation of all residues during the simulation and the root mean square fluctuation (RMSF) of CTSL during the binding of CTSL-control, CTSL-ZINC4097985, and CTSL-ZINC4098355 were plotted as a function of CTSL residue numbers. The CTSL-control and CTSL-ZINC4098355 backbones exhibited consistent fluctuations in the CTSL binding pocket, most likely due to differing orientations, and CTSL-ZINC4097985 showed high fluctuation in several regions such as 38–45, 58–61, and 110–115 residues (Figure 3C). Conversely, the CTSL-control and CTSL-ZINC4098355 complexes had the overall fewest fluctuations. Collectively, ZINC4098355 was more stable than ZINC4097985, and it follows the control RMSF pattern with CTSL.

In order to acquire a deeper understanding of the intricate compactness profile within a biological system, the radius of gyration (Rg) was used. The complexes CTSL-control, CTSL-ZINC4097985, and CTSL-ZINC4098355 exhibited average Rg values of 1.66 nm, 1.68 nm, and 1.64 nm, respectively. The Rg plot revealed that the CTSL-control and CTSL-ZINC4097985 complexes were less compact than the CTSL-ZINC4098355 complex. It was inferred that after the binding of CTSL-control and CTSL-ZINC4097985 compounds, CTSL becomes more stable due to the higher unfolding of CTSL’s native structure (Figure 3D).

**Figure 4:**
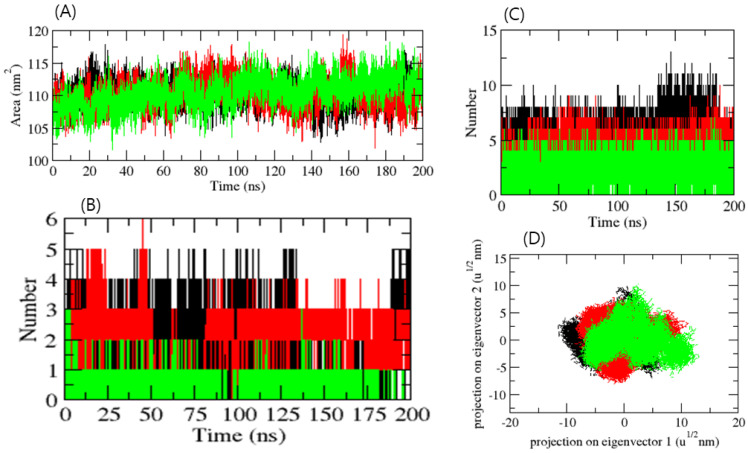
SASA plot of complexes (**A**), number of H-bonds in complexes (**B**), number of H-bonds between ligands and water molecules (**C**), and 2D projection of protein ligand complexes (**D**). Red, green, and black color represent ZINC4097985, ZINC4098355, and control.

A protein’s solvent-accessible surface area (SASA) refers to the extent of its surface that is engaged in interactions with the surrounding solvent molecules. The average SASA values for the three complexes, CTSL-control, CTSL-ZINC4097985, and CTSL-ZINC4098355, were graphed. SASA values for the CTSL-control, CTSL-ZINC4097985, and CTSL-ZINC4098355 complexes were 114.01, 116.12, and 109.23 nm^2^, respectively (Figure 4A). SASA analysis showed that the binding of ZINC4098355 reduced surface exposure, while the control and ZINC4097985 compounds enhanced the surface area of solvent accessibility. The selected docked complexes were also subjected to hydrogen bond analysis. The control and ZINC4097985 exhibited an average 3–5 H-bond with CTSL protein, while the ZINC4098355 compound demonstrated a 1–3 H-bond. In addition, they also formed more H-bonds with solvents in the system. It was inferred that the control and ZINC4097985 compounds interacted more with water molecules in the system, while ZINC4098355 interacted less with water. Therefore, it binds more stably in the catalytic pocket of the CTSL protein (Figure 4B,C). Further, 2D plot analysis of complexes showed that all the complexes overlap very well with each other (Figure 4D).

**Figure 5:**
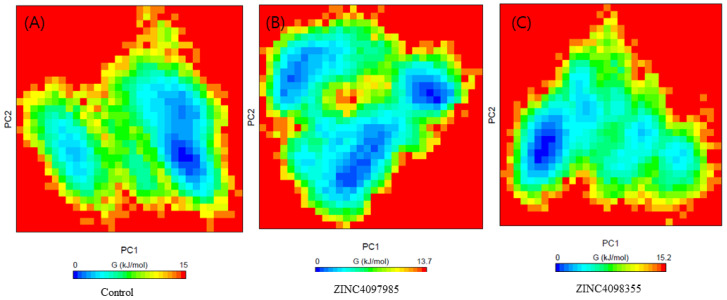
Free energy landscape of complex with CTSL protein. CTSL-control (**A**), CTSL-ZINC4097985 (**B**), and CTSL-ZINC4098355 (**C**).

The free energy landscape of CTSL-control, CTSL-ZINC4097985, and CTSL-ZINC4098355 complexes has been demonstrated in Figure 5A–C. The complex CTSL-control and CTSL-ZINC4098355 formed a similar pattern to CTSL-ZINC4097985. The complex CTSL-ZINC4097985 showed a higher trajectory distribution than the other compounds.

Because of their rich chemical diversity, natural products represent a valuable reservoir of diverse bioactive compounds with promising medicinal potential [25]. Over the past few decades, considerable research endeavors have been dedicated to the identification and extraction of novel natural products from a variety of organisms, such as plants, microorganisms, and other biological entities. Numerous anti-cancer pharmaceuticals have been developed as a result of extensive research into the anti-cancer properties of these naturally occurring compounds and the underlying mechanisms of their action. Interestingly, it is estimated that from 1981 to 2019, about 25% of newly approved anti-cancer medications had natural constituents as their source [26]. Notably, the hits identified in this study are natural compounds that have been demonstrated to bind strongly to the CTSL protein; further investigation could confirm that these substances are potent CTSL inhibitors.

## 3. Methods

### 3.1. Machine Learning Model Building and Screening Natural Compounds

Compound datasets of reported active and inactive CTSL ligands were retrieved from freely available cheminformatics databases, such as ChEMBL. To distinguish between active and inactive compounds, the largest experimental datatype for the CTSL enzyme was used. The activity cutoff for biochemical or biophysical activity (IC_50_) was 1000 nM, and anything above that was considered inactive. Further, molecular fingerprints (FPs) for the active and inactive compounds were determined using the cheminformatics package RDKit (5 March 2022). We counted all circular fragments from each chosen center-heavy atom up to the specified radius of two atoms and 1024 binary bits using the Morgan FPs [27].

Next, the ML models were built with the default Python (3.9) libraries. The classic ML algorithm (RF) method was chosen. The scikit-learn (1.7.3) [28] package is used to implement the RF model with a dataset splitting ratio of 4:1 (train and test). Ten-fold cross-validation was used to assess the trained models, and receiver operating characteristic curves were plotted for RF models. The trained model was then used to screen the natural compound library (Biopurify and Targetmol). Furthermore, we choose the top virtual hits from the natural compound database with a prediction score > 0.6 for further screening using structure-based methods.

### 3.2. CTSL Protein Retrieval and Preparation

The 3D structure of the CTSL protein (PDB ID: 3HHA) was obtained in pdf format from the Protein Data Bank. The structure exhibits a monomeric arrangement, with AZ12878478 as a co-crystal ligand. The co-crystal ligand and water molecules were eliminated from the structure using Discovery Studio (DS) 2021. The ‘clean’ protein was then prepared by employing the “prepared protein” protocol in DS 2021, and the resulting protein structure was saved in pdb format.

### 3.3. Structure-Based Virtual Screening

SBVS is a well-established computational technique used in computer-assisted drug design. This screening methodology is critical to accelerating the process of novel drug design by identifying potentially potent compounds. Specifically, molecular docking-based vs. is an effective method for identifying active compounds from large ligand databases. This is accomplished by assessing the binding affinities of proteins and ligands, which aids in the selection of compounds with promising interactions for further investigation [29]. The PyRx tool (0.8 version) was used to screen the compounds identified by the ML model against the active site of CTSL. The coordinates for the grid center of the CTSL were defined as follows: X = 8.674536, Y = 8.057071, and Z = −2.836607.

### 3.4. Physiochemical and ADMET Properties Prediction

The physicochemical and molecular properties of the top 13 compounds were estimated by the SwissADME web tool (http://www.swissadme.ch/, accessed on 10 July 2023). The ADMET properties of the final top two compounds were predicted using online tools (https://ai-druglab.smu.edu/admet, accessed on 10 July 2023). 

### 3.5. Molecular Dynamics (MD) Simulation

GROMACS 2022 was used to conduct MD simulations of CTSL-control, CTSL-ZINC4097985, and CTSL-ZINC4098355 complexes at 300 K [30]. CTSL enzyme topology was determined using the AMBER 99SB force field [31]. Force-field parameters and lead compound topologies were generated using the AnteChamber server [32]. The lead compound’s atoms were assembled into a complex using the topology of the CTSL enzyme. The CTSL-compound complex was then solvated using the TIP3P water model in a cubic box [33]. To ensure a physiological concentration of 0.15 M, the charges on CTSL-control, CTSL-ZINC4097985, and CTSL-ZINC4098355 were restored by adding Na^+^ and Cl^−^ ions through the gmx genion module. The steepest descent method, with 1500 steps, was used to minimize each system. The temperature was stabilized using the V-rescale thermostat after the system was first brought to equilibrium using an NVT ensemble with a constant number of particles, volume, and temperature of 300 K for 100 ps. The Parrinello-Rahman barostat was then used to equalize the pressure in each system to 1.0 bar [34]. To calculate long-range electrostatic interactions, each equilibrated system was simulated for 200 ns using particle mesh Ewald. The Berendsen (V-rescale) thermostat [35] and the Parrinello-Rahman barostat were used during the simulation to keep the temperature at 300 K and the pressure at 1.0 bar, respectively. GROMACS analysis modules were used to study subsequent trajectories. VMD [36] and PyMOL were used to generate graphical representations of the 3D models.

## 4. Conclusions

This study used a novel approach that combined ML and SBVS techniques to identify potential natural CTSL inhibitors, the expression of which is known to be abnormal in a variety of cancer types. ZINC4097985 and ZINC4098355 have been found to possess strong affinities and specificities for the CTSL binding site, as well as drug-like properties. In a 200 ns MD simulation, these compounds also demonstrated stable binding in complexes with the CTSL protein. ZINC4097985 and ZINC4098355 exhibit potential as viable inhibitors of CTSL, thereby offering therapeutic value in the management of cancer. Nevertheless, it is necessary to conduct experimental validation in order to optimize them as inhibitors of CTSL.

## Figures and Tables

**Table 1:** Binding energies and physicochemical properties of the top 13 compounds.

Compound ID	Binding Affinity (kcal/mol)	RF_Prediction Score	MolWt	MolLogP	HA	HD	RB
ZINC4097985	−7.9	0.794152879	408.403	−0.3084	9	4	4
ZINC4098355	−7.6	0.788373218	422.474	2.6621	8	0	8
ZINC96023730	−6.4	0.684678299	484.721	6.6687	4	1	10
ZINC38429839	−7.2	0.652458095	456.711	6.6289	3	2	5
ZINC13383393	−6.5	0.651968775	358.302	2.2031	6	5	4
ZINC299817526	−6.6	0.648805457	468.678	7.4357	2	2	9
ZINC4098425	−7.5	0.646199152	232.235	0.969	4	1	1
ZINC5665355	−6.6	0.643019115	470.694	7.6597	2	2	9
ZINC1702729	−6.9	0.640874436	388.416	2.8323	7	3	6
ZINC238760072	−6.5	0.633125885	376.493	4.5247	5	0	8
ZINC238790964	−6.4	0.624866697	430.541	2.3323	6	3	3
ZINC28876559	−6.4	0.621405139	440.712	7.2275	2	2	5
ZINC3982483	−6.6	0.620906903	406.545	−0.857	8	5	7
AZ12878478 (control)	−6.3	-	-	-	-	-	-

No. of H acceptors: HA; No. of H donors: HD; No. of rotatable bonds: RB.

**Table 2:** ADMET prediction for the top two compounds, ZINC4097985 and ZINC4098355.

Molecule Property		Value		Unit
		ZINC4097985	ZINC4098355	
**Absorption**				
Caco-2 Permeability		−5.51	−5.27	log(cm/s)
HIA		62.85	66.63	%
Pgp Inhibition		36.88	30.23	%
log D7.4		1.83	1.72	log-ratio
Aqueous Solubility		−4.46	−4.09	log(mol/L)
Oral Bioavailability		36.66	40.58	%
**Distribution**				
BBB		18.77	29.17	%
PPBR		35.01	45.2	%
VDss		3.13	3.39	L/kg
**Metabolism**				
CYP2C9	Inhibition	50.93	53.89	%
CYP2D6		98.02	92.92	
CYP3A4		34.92	35.24	
CYP2C9	Substrate	34.14	32.66	
CYP2D6		51.03	56.54	
CYP3A4		41.78	42.75	
**Excretion**				
Half-Life		58.17	63.46	h
CL-Hepa		47.36	55.3	μL min^−1^ (106 cells)^−1^
CL-Micro		40.61	48.28	mL min^−1^ g^−1^
**Toxicity**				
hERG Blockers		43.62	40.04	%
Ames		48.32	39.31	
DILI		49.79	59.98	
LD50		2.75	2.45	−log(mol/kg)

## Data Availability

The data presented in this study are available in this article.

## References

[1] Turk V., Stoka V., Vasiljeva O., Renko M., Sun T., Turk B., Turk D. (2012). Cysteine cathepsins: From structure, function and regulation to new frontiers. Biochim. Biophys. Acta.

[2] Pu J., Guardia C.M., Keren-Kaplan T., Bonifacino J.S. (2016). Mechanisms and functions of lysosome positioning. J. Cell Sci..

[3] Fonovic M., Turk B. (2014). Cysteine cathepsins and extracellular matrix degradation. Biochim. Biophys. Acta.

[4] Kukor Z., Mayerle J., Kruger B., Toth M., Steed P.M., Halangk W., Lerch M.M., Sahin-Toth M. (2002). Presence of cathepsin B in the human pancreatic secretory pathway and its role in trypsinogen activation during hereditary pancreatitis. J. Biol. Chem..

[5] Reiser J., Adair B., Reinheckel T. (2010). Specialized roles for cysteine cathepsins in health and disease. J. Clin. Investig..

[6] Gocheva V., Joyce J.A. (2007). Cysteine cathepsins and the cutting edge of cancer invasion. Cell Cycle.

[7] Sudhan D.R., Siemann D.W. (2013). Cathepsin L inhibition by the small molecule KGP94 suppresses tumor microenvironment enhanced metastasis associated cell functions of prostate and breast cancer cells. Clin. Exp. Metastasis.

[8] Rudzinska M., Parodi A., Soond S.M., Vinarov A.Z., Korolev D.O., Morozov A.O., Daglioglu C., Tutar Y., Zamyatnin A.A. (2019). The Role of Cysteine Cathepsins in Cancer Progression and Drug Resistance. Int. J. Mol. Sci..

[9] Patel S., Homaei A., El-Seedi H.R., Akhtar N. (2018). Cathepsins: Proteases that are vital for survival but can also be fatal. Biomed. Pharmacother..

[10] Hashimoto Y., Kondo C., Kojima T., Nagata H., Moriyama A., Hayakawa T., Katunuma N. (2006). Significance of 32-kDa cathepsin L secreted from cancer cells. Cancer Biother. Radiopharm..

[11] Rofstad E.K., Mathiesen B., Kindem K., Galappathi K. (2006). Acidic extracellular pH promotes experimental metastasis of human melanoma cells in athymic nude mice. Cancer Res..

[12] Skrzydlewska E., Sulkowska M., Koda M., Sulkowski S. (2005). Proteolytic-antiproteolytic balance and its regulation in carcinogenesis. World J. Gastroenterol..

[13] Sobotic B., Vizovisek M., Vidmar R., Van Damme P., Gocheva V., Joyce J.A., Gevaert K., Turk V., Turk B., Fonovic M. (2015). Proteomic Identification of Cysteine Cathepsin Substrates Shed from the Surface of Cancer Cells. Mol. Cell Proteom..

[14] Gocheva V., Zeng W., Ke D., Klimstra D., Reinheckel T., Peters C., Hanahan D., Joyce J.A. (2006). Distinct roles for cysteine cathepsin genes in multistage tumorigenesis. Genes. Dev..

[15] Chauhan S.S., Goldstein L.J., Gottesman M.M. (1991). Expression of cathepsin L in human tumors. Cancer Res..

[16] Xu X., Yu T., Dong L., Glauben R., Wu S., Huang R., Qumu S., Chang C., Guo J., Pan L. (2023). Eosinophils promote pulmonary matrix destruction and emphysema via Cathepsin L. Signal Transduct. Target. Ther..

[17] Sadybekov A.V., Katritch V. (2023). Computational approaches streamlining drug discovery. Nature.

[18] Talele T.T., Khedkar S.A., Rigby A.C. (2010). Successful applications of computer aided drug discovery: Moving drugs from concept to the clinic. Curr. Top. Med. Chem..

[19] Marquis R.W., James I., Zeng J., Trout R.E., Thompson S., Rahman A., Yamashita D.S., Xie R., Ru Y., Gress C.J. (2005). Azepanone-based inhibitors of human cathepsin L. J. Med. Chem..

[20] Kuhn B., Tichy M., Wang L., Robinson S., Martin R.E., Kuglstatter A., Benz J., Giroud M., Schirmeister T., Abel R. (2017). Prospective Evaluation of Free Energy Calculations for the Prioritization of Cathepsin L Inhibitors. J. Med. Chem..

[21] Parker E.N., Song J., Kishore Kumar G.D., Odutola S.O., Chavarria G.E., Charlton-Sevcik A.K., Strecker T.E., Barnes A.L., Sudhan D.R., Wittenborn T.R. (2015). Synthesis and biochemical evaluation of benzoylbenzophenone thiosemicarbazone analogues as potent and selective inhibitors of cathepsin L. Bioorg. Med. Chem..

[22] Siklos M., BenAissa M., Thatcher G.R. (2015). Cysteine proteases as therapeutic targets: Does selectivity matter? A systematic review of calpain and cathepsin inhibitors. Acta Pharm. Sin. B.

[23] Fleming F.F., Yao L., Ravikumar P.C., Funk L., Shook B.C. (2010). Nitrile-containing pharmaceuticals: Efficacious roles of the nitrile pharmacophore. J. Med. Chem..

[24] Tian H., Ketkar R., Tao P. (2022). ADMETboost: A web server for accurate ADMET prediction. J. Mol. Model..

[25] Atanasov A.G., Zotchev S.B., Dirsch V.M., Supuran C.T., The International Natural Product Sciences Taskforce (2021). Natural products in drug discovery: Advances and opportunities. Nat. Rev. Drug Discov..

[26] Newman D.J., Cragg G.M. (2020). Natural Products as Sources of New Drugs over the Nearly Four Decades from 01/1981 to 09/2019. J. Nat. Prod..

[27] Zagidullin B., Wang Z., Guan Y., Pitkanen E., Tang J. (2021). Comparative analysis of molecular fingerprints in prediction of drug combination effects. Brief. Bioinform..

[28] Abraham A., Pedregosa F., Eickenberg M., Gervais P., Mueller A., Kossaifi J., Gramfort A., Thirion B., Varoquaux G. (2014). Machine learning for neuroimaging with scikit-learn. Front. Neuroinform..

[29] Zhang B., Li H., Yu K., Jin Z. (2022). Molecular docking-based computational platform for high-throughput virtual screening. CCF Trans. High. Perform. Comput..

[30] Van Der Spoel D., Lindahl E., Hess B., Groenhof G., Mark A.E., Berendsen H.J. (2005). GROMACS: Fast, flexible, and free. J. Comput. Chem..

[31] Showalter S.A., Bruschweiler R. (2007). Validation of Molecular Dynamics Simulations of Biomolecules Using NMR Spin Relaxation as Benchmarks: Application to the AMBER99SB Force Field. J. Chem. Theory Comput..

[32] Sousa da Silva A.W., Vranken W.F. (2012). ACPYPE–AnteChamber PYthon Parser interfacE. BMC Res. Notes.

[33] Yagasaki T., Matsumoto M., Tanaka H. (2020). Lennard-Jones Parameters Determined to Reproduce the Solubility of NaCl and KCl in SPC/E, TIP3P, and TIP4P/2005 Water. J. Chem. Theory Comput..

[34] Mencel K., Starynowicz P., Siczek M., Piecha-Bisiorek A., Jakubas R., Medycki W. (2019). Symmetry breaking structural phase transitions, dielectric properties and molecular motions of formamidinium cations in 1D and 2D hybrid compounds: (NH_2_CHNH_2_)_3_[Bi_2_Cl_9_] and (NH_2_CHNH_2_)_3_[Bi_2_Br_9_]. Dalton Trans..

[35] Mor A., Ziv G., Levy Y. (2008). Simulations of proteins with inhomogeneous degrees of freedom: The effect of thermostats. J. Comput. Chem..

[36] Humphrey W., Dalke A., Schulten K. (1996). VMD: Visual molecular dynamics. J. Mol. Graph..

